# The diversity of well-being indicators: a latent profile analysis

**DOI:** 10.3389/fpsyg.2024.1304074

**Published:** 2024-03-04

**Authors:** Calen J. Horton, Lisa C. Walsh, Anthony Rodriguez, Victor A. Kaufman

**Affiliations:** ^1^Arkoda Research Group, Anchorage, AK, United States; ^2^Department of Psychology, University of California, Los Angeles, Los Angeles, CA, United States; ^3^RAND Corporation, Santa Monica, CA, United States

**Keywords:** subjective well-being, happiness, life satisfaction, positive affect, negative affect, latent profile analysis, domain satisfaction, couple satisfaction

## Abstract

**Introduction:**

Research on the dimensional structure of subjective well-being (SWB) suggests a five-dimensional solution, consisting of the three established dimensions of life satisfaction, positive affect, and negative affect, and two additional empirically supported dimensions: domain satisfaction and happiness. While these dimensions can be aggregated into a superordinate SWB construct, little research has explored how these dimensions differ in their variation across subpopulations of individuals.

**Methods:**

The present study addresses this gap via secondary analysis of a sample of 1,487 partnered individuals, using the five dimensions of SWB as indicators for latent profile analysis.

**Results:**

Analyses returned five profiles, which we labeled Satisfied, Ambivalent, Indifferent, Dissatisfied, and Very Dissatisfied. In the Ambivalent and Indifferent profiles, the dimensions of negative affect and happiness exhibit discrepant behavior, resulting in shape differences. The five profiles are organized with reference to the external criterion of couple satisfaction. At the theoretical level, the results of the present study have the potential to inform current debates about the structure of well-being.

**Discussion:**

These findings suggest that, while SWB can usually be measured as a unidimensional construct, there is still merit to using multidimensional approaches and alternative forms of measurement—such as LPA—that capture complexities normally absent from unidimensional treatments. At the practical level, the results of the current study have the potential to inform well-being interventions (both clinical and otherwise), suggesting that those dealing with well-being in real life situations should pause before concluding that the absence of negativity implies the presence of positivity, or vice versa.

## 1 Introduction

In his seminal paper on Subjective Well-Being (SWB), Diener (1984) proposed an initial tripartite structure for SWB consisting of three subdimensions: life satisfaction, positive affect, and negative affect. Ever since then, researchers have disagreed over the structure of SWB. One point of disagreement is the content of the subdimensions—some researchers believe there are additional subdimensions, while others readily use a single subdimension (usually life satisfaction or positive affect) as a proxy for the larger SWB construct. A second, more fundamental disagreement involves treatment of the subdimensions—should they be aggregated to form a single SWB score? Or should they be studied separately (Busseri and Sadava, 2011)? This is important because, as Busseri and Sadava (2011) have noted, one implication of a model where SWB dimensions can be aggregated is that the positive and negative aspects of SWB are presumed to be univariate–that is, a simplified bipolar model anchored by positivity at one end and negativity at the other. The other possibility is that the positive and negative aspects are separable, being either bivariate or at least containing aspects which cannot be captured by a simple univariate model (see Schimmack, 2008). At present this question is still actively discussed (e.g., Iasiello and Van Agteren, 2020; Zhao and Tay, 2023).

Recent research (e.g., Busseri, 2018; Kaufman et al., 2022a) suggests SWB is multidimensional, with subdimensions that contribute to a superordinate SWB construct. This lends credence to aggregating the subdimensions. In our own research (see Kaufman et al., 2022a) we examined the three subdimensions originally proposed by Diener (1984) and two additional constructs: The first, domain satisfaction, addresses satisfaction in specific areas of life (e.g., work, family) instead of global evaluations. The second, happiness, is thought to be an especially SWB-relevant emotion, but was left out of the most common measure of positive affect, the Positive and Negative Affect Schedule (PANAS; see Watson et al., 1988). Our research found that while all five constructs can be considered subdimensions of SWB, ultimately SWB can be considered *essentially unidimensional*, meaning the subdimensions can be aggregated to form a single score.

However, an important question remains unaddressed: how do the five subdimensions behave in terms of their variation across subpopulations? One danger of establishing that the components of SWB can be aggregated is that some may conclude that the subdimensions are no longer worth studying by themselves. Examining variations across subpopulations is a good test of this assumption; if SWB subdimensions are simple, passive reflections of the larger SWB construct, we might expect them to rise and fall in tandem with each other across subgroups of a sample, exhibiting little by way of idiosyncratic behavior.

Alternatively, the relationship between subdimensions of SWB may be more complex. If so, one might expect them to exhibit unique patterns of variation across subgroups, combining in ways that could not be predicted if the relationship between the subdimensions was simple and direct. If this is the case, it would imply that the subdimensions are still worthy of further study by themselves. The present study addresses this possibility using latent profile analysis (LPA), which is ideal for studying variation across subgroups.

We start by reviewing the internal conceptual divisions within SWB, as these support the argument that we might expect complex variations of subdimensions across subgroups.

### 1.1 Distinctions between SWB dimensions

There are two major distinctions made between subdimensions of SWB; the division between its cognitive and affective components, and the further division of affect into positive and negative components. An additional minor distinction involves happiness, which seems to contain elements of both cognition and affect.

The internal division in cognition was initially highlighted by Diener (1984), who differentiated between global life satisfaction, which measures cognitive evaluations of life as a whole, and domain satisfaction, which measures cognitive evaluations of specific areas of life such as work, relationships, and spirituality. Diener et al. (1999) recommended separating the two, though Schimmack (2008) has noted they are closely related, with correlations up to *r* = 0.70.

The affective component appears more complex. Positive and negative affect are often treated as a univariate construct (e.g., when a person feels good about their day it implies they do not feel bad about their day). However, evidence suggests they can also be treated as bivariate (e.g., a person can be frequently happy and also frequently sad, or experience very little of either emotion; see Schimmack, 2008). As Schimmack noted, the separability of positive and negative affect is influenced by factors like question content, temporal duration, and others, but even accounting for those, positive and negative affect remain distinct.

The separability of positive and negative affect has parallels elsewhere. For example, Rogge et al. (2017) demonstrated that positive and negative relationship evaluations can be treated as a bivariate scale with separable dimensions. And researchers are actively exploring whether well-being and its opposite, ill-being, should similarly be treated as separable (e.g., Iasiello and Van Agteren, 2020; Zhao and Tay, 2023).

Finally, research suggests that happiness contains elements of both cognition and affect. Happiness is often considered the quintessential positive emotion, but as Schimmack (2003) noted, it is absent from the PANAS scale developed by Watson et al. (1988), which is typically used to measure positive affect. To address the gap, Lyubomirsky and Lepper (1999) developed the Subjective Happiness Scale (SHS). However, the SHS taps global, cognitive appraisals; unlike emotion measures such as the PANAS, which ask people to report the frequency or intensity of specific emotions over a time interval, the SHS asks people to evaluate their general happiness, and also to situate their happiness in comparison with others.

Even when not measured using the SHS, happiness may be more cognitively relevant than other emotions; in an experience sampling study, Schimmack (2003) found that participants’ life satisfaction correlated higher with self-reports of happiness than all other positive emotions, suggesting that happiness overlaps more with cognitive variables like life satisfaction.

Additional research suggests that happiness is subject to cognitive modifiers; one such modifier is the fear of happiness (Joshanloo, 2013)—the belief that happiness can potentially lead to bad outcomes (see Yildirim, 2019, for a study of the downstream effects of fear of happiness on well-being). A second cognitive modifier is the externality of happiness, or the belief that happiness is uncontrollable and primarily caused by external variables (see Yildirim and Belen, 2019). Fear of happiness has been shown to predict both emotional and psychological aspects of well-being above and beyond personality variables, such as the activity of the Behavioral Inhibition System and Behavioral Activation System (Yildirim and Belen, 2018), suggesting that the cognitive component of happiness is consequential.

In practice, the separability of these different aspects of well-being—the division of affect into positive and negative components, as well as the division of SWB as a whole (and happiness in particular) into both cognitive and affective components—suggests that the dimensions comprising SWB may be subject to complicated internal dynamics which merit further inquiry.

### 1.2 Latent profile analysis

Given the differences between the subdimensions comprising SWB it is reasonable to expect that the relationships between them may be complex even if they can be aggregated to form a single SWB score. In the present study we sought to model these relationships. Latent profile analysis (LPA), which can model complex patterns of interaction between variables parsimoniously by representing them as groups (called “profiles”), is ideal for this investigation (see Spurk et al., 2020). We therefore used measures of the five subdimensions of SWB as indicator variables for LPA, to identify subgroups within our sample, determine their magnitude, and explore their differences.

Latent profile analysis can return two types of results, referred to as level differences and shape differences between profiles. To offer a simplified case, imagine asking volunteers two questions – how happy their day was, and how social their day was. If a profile analysis of these volunteers returned three groups – one with high levels of both happiness and sociality, one with medium, and one with low, those would constitute level differences between profiles, where levels of indicators vary directly (or inversely) with each other across subgroups. Level differences indicate a simple, direct relationship between the two variables, and researchers may conclude that happiness and sociality are related in an uncomplicated way.

However, if the same analysis returned an extra group – one with very low levels of sociality and high levels of happiness, for example, breaking the norm – that profile would be said to have a shape difference, and would indicate a more complex relationship between the variables meriting further inquiry. Based on our review of the literature, we anticipate that LPA will reveal shape differences in profiles, in addition to normal level differences. We remain agnostic regarding which variables, specifically, will drive such shape differences.

When LPA is conducted it is considered best practice to arrange the resulting profiles according to their scores on a relevant outcome variable (Spurk et al., 2020). This serves two purposes; the first purpose of such an outcome is to establish the discriminant validity of the profiles by demonstrating that they each predict different levels of the outcome variable. The second purpose is to situate the profile solution inside of the larger nomological network of constructs related to the indicators.

In our case, our indicator variables were all subdimensions of SWB. Therefore it was important to pick an external criterion variable that is theoretically important relative to SWB. As such, in the present study, we selected the Couples Satisfaction Index (CSI; Funk and Rogge, 2007) as an outcome measure to use for validating the profile solution.

The CSI is a measure of relationship quality focused on a person’s evaluation of their partner and of the relationship overall. It makes an ideal criterion variable in the present study for two reasons. First, romantic relationship satisfaction is widely considered to be one of the most important predictors of SWB (see Proulx et al., 2007, for a meta-analytic review of the relationship between the two). Second, research conducted in the past ten years suggests that romantic relationship satisfaction may also be a complex construct. While it is traditionally treated as univariate, anchored on one end by positive evaluations and on the other by negative, research by Rogge et al. (2017) suggests that relationship satisfaction may also contain nuances that emerge when positive and negative relationship qualities are treated as bivariate dimensions.

While the original CSI developed by Funk and Rogge (2007) is a univariate measure, the later research conducted by Rogge et al. (2017) suggests that the construct of relationship satisfaction as a whole may be fruitful ground for the exploration of complex relationships between the positive and negative aspects of well-being. By taking such a broad and consequential relationship—one traditionally considered to share a linear relationship with SWB—and using the indicators to explore whether the relationship is more complex and marked by profile arrangements with shape differences, LPA may contribute new knowledge to the understanding of the relationship between SWB and couple satisfaction.

## 2 Methods

### 2.1 Participants and procedures

We conducted a secondary analysis of a preexisting dataset (see Kaufman et al., 2022b). A nationally representative sample of U.S. residents was recruited (via Dynata), with informed consent, to participate in an online survey. The initial sample consisted of 3,699 people who were further filtered using five attention-check questions to ensure that participants were engaged with the study. Participants were only included if they passed all five attention checks. This reduced the sample to 2,000. This was further trimmed to 1,487 participants who indicated that they were in a relationship; single participants were excluded in this study because the outcome variable used to organize profiles was couple satisfaction. The participants were near-evenly split between males (49.7%) and females (50.3%). Regarding race and ethnicity, the sample consisted of 68.3% White/Caucasian participants, 17.2% Hispanic, 10.6% Black, 4.6% Asian, and 1.6% who classified themselves as “Other.” In terms of education and socioeconomic status, 62% of participants had at least a college degree and 59.3% reported an income of $75,000 or higher. Participant demographics differed slightly from the original sample which included people who were married or in relationships; participants in the present study tended to be more wealthy and more educated. The demographics for the full sample of singles in the present study can be seen in Table 1. The demographics for the original sample can be seen in Kaufman et al. (2022b, Study 2).

**Table 1:** Participant demographics for full sample and individual profiles.

	Full Sample		Satisfied		Ambivalent		Indifferent		Dissatisfied		Very dissatisfied	
Characteristic	*N*	%	*n*	%	*n*	%	*n*	%	*n*	%	*n*	%
*N/n*	1,487	100	664	44.7	125	8.4	373	25.1	188	12.6	137	9.2
Mean age	*M* = 45.1		*M* = 47.8		*M* = 40.9		*M* = 46.4		*M* = 40.3		*M* = 39.1	
*Age by category*												
18–24	135	9.1%	38	5.7%	21	16.8%	24	6.4%	33	17.6%	19	13.9%
25–34	273	18.4%	87	13.1%	35	28.0%	67	18.0%	47	25.0%	37	27.0%
35–44	294	19.8%	124	18.7%	16	12.8%	84	22.5%	32	17.0%	38	27.7%
45–54	310	20.8%	138	20.8%	29	23.2%	79	21.2%	38	20.2%	26	19.0%
55–64	285	19.2%	159	23.9%	16	12.8%	76	20.4%	24	12.8%	10	7.3%
65+	190	12.8%	118	17.8%	8	6.4%	43	11.5%	14	7.4%	7	5.1%
*Gender*												
Male	739	49.7%	344	51.8%	50	40.0%	212	56.8%	79	42.0%	54	39.4%
Female	748	50.3%	320	48.2%	75	60.0%	161	43.2%	109	58.0%	83	60.6%
Race/Ethnicity*												
White	1,015	68.3%	471	70.9%	86	68.8%	247	66.2%	121	64.4%	90	65.7%
Black	157	10.6%	70	10.5%	20	16.0%	35	9.4%	18	9.6%	14	10.2%
Hispanic	256	17.2%	100	15.1%	17	13.6%	74	19.8%	39	20.7%	26	19.0%
Asian	69	4.6%	26	3.9%	4	3.2%	22	5.9%	9	4.8%	8	5.8%
Other	24	1.6%	10	1.5%	4	3.2%	5	1.3%	4	2.1%	1	0.7%
*Education*												
<High School	22	1.5%	3	0.5%	3	2.4%	5	1.3%	4	2.1%	7	5.1%
High School Grad	209	14.1%	70	10.5%	17	13.6%	42	11.3%	46	24.5%	34	24.8%
Some College	330	22.2%	124	18.7%	30	24.0%	88	23.6%	47	25.0%	41	29.9%
College Degree	591	39.7%	285	42.9%	45	36.0%	162	43.4%	64	34.0%	35	25.5%
Graduate Degree	331	22.3%	180	27.1%	29	23.2%	75	20.1%	27	14.4%	20	14.6%
Prefer not to answer	4	0.3%	2	0.3%	1	0.8%	1	0.3%	0	0%	0	0%
*Income*												
<$30,000	172	11.6%	33	5.0%	16	12.8%	46	12.3%	32	17.0%	45	32.8%
$30,000–$49,999	196	13.2%	57	8.6%	20	16.0%	55	14.7%	35	18.6%	29	21.2%
$50,000 - $74,999	238	16.0%	111	16.7%	18	14.4%	59	15.8%	28	14.9%	22	16.1%
$75,000 - $99,999	245	16.5%	120	18.1%	20	16.0%	61	16.4%	30	16.0%	14	10.2%
$100,000–$149,999	306	20.6%	162	24.4%	25	20.0%	72	19.3%	31	16.5%	16	11.7%
>$150,000	330	22.2%	181	27.3%	26	20.8%	80	21.4%	32	17.0%	11	8.0%

*Race/ethnicity categories were not mutually exclusive.

### 2.2 Measures

#### 2.2.1 Life satisfaction

This was measured using the Satisfaction with Life Scale (SWLS; Diener et al., 1985; 5 items; Cronbach’s α = 0.90; 1 = *completely disagree* to 6 = *completely agree*; e.g., “In most ways my life is close to ideal”). The SWLS is a highly cited measure of life satisfaction, and it has been extensively validated (e.g., Pavot et al., 1991).

#### 2.2.2 Domain satisfaction

Domain satisfaction was measured using the Personal Wellbeing Index (PWI; Tomyn et al., 2013; 8 items; Cronbach’s α = 0.93; 1 = *No satisfaction at all* to 6 = *completely satisfied;* e.g., “How satisfied are you with your standard of living?”). The PWI assesses satisfaction with eight life domains—standard of living, personal health, life achievement, personal relationships, personal safety, community connectedness, future security, and spirituality. It has also received extensive validation across multiple samples and populations (see Tomyn et al., 2013).

#### 2.2.3 Happiness

Happiness was measured using the Subjective Happiness Scale (SHS; Lyubomirsky and Lepper, 1999; 4 items; Cronbach’s α = 0.84; 1 = *Less happy* to 7 = *More happy*; e.g., “Compared to my peers, I consider myself [less happy/more happy]”). The SHS is a highly cited and well-validated measure (for validation, see Lyubomirsky and Lepper, 1999).

#### 2.2.4 Positive affect

To assess positive affect, we used items from the International Personality Item Pool scale for Joyfulness (IPIP, Goldberg, 2019; 3 items; Cronbach’s α = 0.76; 1 = *Very inaccurate* to 5 = *Very accurate;* e.g., “Have a lot of fun”). The IPIP Joyfulness scale consists of ten items; only five of which reflected positivity. Of those five, we ultimately kept the three items that referenced either specific emotions (e.g., “Radiate joy”) or general positive affect (e.g., “Feel lucky most of the time”). The three-question measure of positive affect has been used previously (see Kaufman et al., 2022a) and has demonstrated adequate psychometric properties.

#### 2.2.5 Negative affect

To assess negative affect, we selected items from the Eysenck Personality Scale for Neuroticism (EPQ; Eysenck and Eysenck, 1993; 4 items; Cronbach’s α = 0.81; 1 = *Yes*, 2 = *No*; e.g., “Do you often feel lonely?”). Items were retained because they denoted negative affect states using the word “feel.” The items chosen assessed misery, feeling “fed up,” dullness, and loneliness. They are comparable to items in other validated measures (e.g., Diener and Emmons, 1984; Harmon-Jones et al., 2016, see also Revord, 2021) assessing sadness, anger, boredom and distress. This 3-item measure was also used previously (see Kaufman et al., 2022a). To aid interpretability, Negative Affect was reverse-scored in the present study; higher NA scores in both the tables and figures indicate lower negative affect.

#### 2.2.6 Couple satisfaction

This was measured using ten items from the Couples Satisfaction Index (CSI; Funk and Rogge, 2007; Cronbach’s α = 0.98; 0 = *Not at all* to 5 = *Completely*; e.g., “How well does your partner meet your needs?”). The CSI’s psychometric properties and validity have been tested extensively (see Funk and Rogge, 2007).

### 2.3 Analytic strategy

All variables were converted to *Z*-scores. We used LPA to identify profiles based on our indicators using Mplus (version 8.1). We ran a series of sequential models ranging from one to six profiles. We determined the best model solution by evaluating information criteria and likelihood ratio tests. Once we found an optimal solution, individuals were assigned profiles based on the greatest probability of group membership. Finally, we used the manual three-step auxiliary BCH approach to test for differences in couple satisfaction using a Wald chi-square test (Asparouhov and Muthén, 2014).

## 3 Results

**Figure 1:**
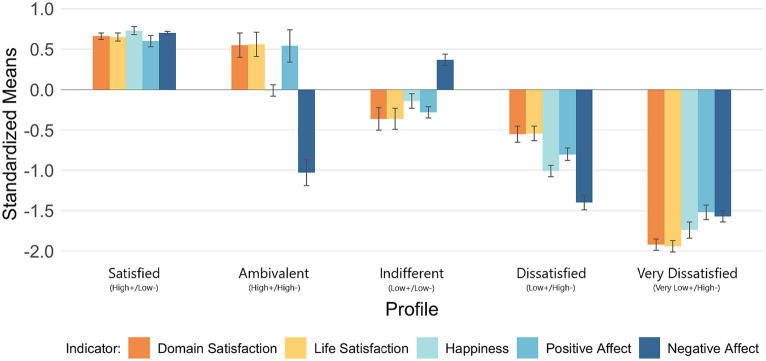
Indicator patterns by profile. Profiles ordered from highest to lowest couple satisfaction. Negative affect was reverse scored; higher scores indicate lower negative affect. Error bars indicate standard errors.

A correlation matrix of all the main study variables is included in the Supplementary materials. All correlations exceeded *r* = 0.41 (*p* < 0.001). Using LPA, we successfully identified heterogeneous groups. Information criteria and likelihood ratio tests suggested the 5-profile model was the optimal solution (see Supplementary materials and Figure 1). The demographic information for the five profiles as well as the main sample can be seen in Table 1.

A follow-up BCH analysis confirmed that the profiles differed in levels of the criterion variable, couple satisfaction (Wald’s *χ*^2^ = 518.46, *p* < 0.001). The results of the BCH analyses provide evidence for the discriminant validity of the profiles (see Table 2). The profiles are analogous to those described by Rogge et al. (2017). Accordingly, we labeled our profiles using similar nomenclature, based on mean levels of positivity and negativity (see Figure 1). Standardized variable means and standard deviations across profiles can be seen in Table 3. Of the five profiles, three of them (Satisfied, Dissatisfied, and Very Dissatisfied) exhibited only level differences, with the positive indicators varying directly with each other, and inversely with the negative indicator. Two profiles (Ambivalent and Indifferent) exhibited more complex shape differences.

**Table 2:** Between-profile tests for differences in couple satisfaction.

	Couple satisfaction	
	*M*	*SE*
Profile 1 (Satisfied)	69.90	0.45
Profile 2 (Ambivalent)	63.31	1.44
Profile 3 (Indifferent)	56.47	0.84
Profile 4 (Dissatisfied)	51.52	1.33
Profile 5 (Very Dissatisfied)	38.92	1.87
	Wald *χ*^2^	*p*
Overall	518.46	<0.001
Profile 1 vs. 2	19.16	<0.001
Profile 1 vs. 3	199.02	<0.001
Profile 1 vs. 4	170.39	<0.001
Profile 1 vs. 5	260.51	<0.001
Profile 2 vs. 3	16.92	<0.001
Profile 2 vs. 4	36.20	<0.001
Profile 2 vs. 5	107.34	<0.001
Profile 3 vs. 4	9.88	0.002
Profile 3 vs. 5	73.66	<0.001
Profile 4 vs. 5	30.16	<0.001

**Table 3:** Standardized descriptive statistics by profile.

			Outcome		Indicators									
			Couple satisfaction		Domain satisfaction		Life satisfaction		Positive affect		Negative affect		Happiness	
	*n*	%	*M*	*SE*	*M*	*SE*	*M*	*SE*	*M*	*SE*	*M*	*SE*	*M*	*SE*
Satisfied	664	44.7%	69.90	0.45	0.66	0.04	0.65	0.05	0.60	0.07	0.70	0.02	0.73	0.05
Ambivalent	125	8.4%	63.31	1.44	0.55	0.15	0.56	0.15	0.54	0.20	−1.03	0.16	−0.01	0.07
Indifferent	373	25.1%	56.47	0.84	−0.36	0.14	−0.36	0.13	−0.28	0.07	0.37	0.07	−0.14	0.09
Dissatisfied	188	12.6%	51.52	1.33	−0.55	0.10	−0.54	0.09	−0.80	0.08	−1.40	0.09	−1.01	0.07
Very Dissatisfied	137	9.2%	38.92	1.87	−1.92	0.07	−1.94	0.07	−1.52	0.09	−1.57	0.07	−1.74	0.10

Table shows standardized measures (*Z*-scores) of variables for each group (relative to full sample mean, *M* = 0, *SD* = 1).

Negative affect was reverse scored; higher scores indicate lower negative affect.

Profiles varied substantially in size; the largest profile by a substantial margin was the Satisfied profile, comprising 44.7% of the sample (*n* = 664). The second largest was the Indifferent profile, comprising 25.1% of the sample (*n* = 373). In order, the Dissatisfied (*n =* 188, 12.6%), Very Dissatisfied (*n* = 137, 9.2%) and Ambivalent (*n =* 125, 8.4%) profiles comprised the remainder.

Across profiles, levels of life satisfaction and domain satisfaction tended to rise and fall in tandem with each other, and positive affect closely mirrors these. Only negative affect and happiness behave discrepantly. It appears that negative affect, as the sole negative subdimension of SWB, can either be congruent with the positive subdimensions (e.g., low negativity matching high positivity, or vice versa) or incongruent (e.g., high negativity conflicting with high positivity, or vice versa). Happiness seems to be a compromise; in profiles where negativity and positivity conflict, happiness lies between the two.

As such, the Ambivalent profile exhibits high negative affect (*M* = −1.03, *SE* = 0.16) but also moderately high positive affect (*M* = 0.54, *SE* = 0.20), life satisfaction (*M* = 0.56, *SE* = 0.15), and domain satisfaction (*M* = 0.55, *SE* = 0.15). Happiness levels in the Ambivalent profile lie midway between the positive and negative subdimensions (*M* = −0.01, *SE* = 0.07). The Indifferent profile exhibits low negative affect (*M* = 0.37, *SE* = 0.07) but also low positive affect (*M* = −0.28, *SE* = 0.07), life satisfaction (*M* = −0.36, *SE* = 0.13), and domain satisfaction (*M* = −0.36, *SE* = 0.14). Happiness again lies near the midpoint (*M* = −0.14, *SE* = 0.09).

## 4 Discussion

Of the five profiles, only the Ambivalent and Indifferent profiles show shape differences. However, together, these two profiles comprise 33.5% of the overall sample. Our sample is nationally representative, so our results, if generalized, suggest that many U.S. coupled adults, rather than simply feeling “good overall” or “bad overall,” may experience complex combinations of positive and negative aspects of SWB.

This finding echoes prior research implying the potential usefulness of examining positivity and negativity with bivariate measures. Rogge et al. (2017) found that the two are separable in the domain of relationship evaluations; this study finds a similar pattern in the subdimensions of SWB which manifests as distinct profiles, one (Ambivalent; 8.4% of the sample) marked by higher levels of positivity and negativity, the other (Indifferent; 25.1%) marked by lower positivity and negativity. The primary driver in the formation of the Ambivalent and Indifferent profiles appears to be negative affect, which moves in the opposite direction, relative to three of the positive subdimensions (life satisfaction, domain satisfaction, and positive affect) of SWB. The dimension of happiness appears to be a secondary driver of these differences, occupying a midpoint between negative affect and the rest. These differences are most acute in the Ambivalent profile, where positive and negative scores deviate substantially from the sample mean.

We also note that these findings appear to be robust to a classic confounding factor with regards to the separability of positive and negative affect—emotional arousal. Positive and negative emotions have opposite valence, but both positive and negative emotions can be high arousal (e.g., excited, afraid) or low arousal (e.g., calm, depressed). As Schimmack (2008) noted, the arousal component of positive and negative emotions is likely to correlate positively across emotions (e.g., those who are easily excited are often more likely to be easily angered) even as the valence component correlates negatively (e.g., those prone to sadness are often less prone to happiness). Measures containing many high-arousal items, like the PANAS, may suppress some of the negative correlation associated with valence, increasing the separability of positive and negative affect. Early researchers identified this as a possible alternative explanation for the separability of positive and negative affect (see Schimmack, 2008, for a review).

In the present study, however, our measures were constructed from items that were more balanced in terms of arousal levels, unlike scales that consist primarily of high-arousal items. The present analysis suggests that even with more balanced measures of emotion, negativity and positivity still remain somewhat separable, resulting in complex shape differences between latent profiles. This implies that the shape differences captured by our Indifferent and Ambivalent profiles may be robust across different measures of positive affect and negative affect, though further studies are required.

### 4.1 Contributions of the present research

The present findings have theoretical and practical implications for researchers and practitioners who work with well-being. At the theoretical level our study has the potential to inform current debates about how to measure and conceptualize SWB. The debate over whether the positive and negative aspects of SWB should be treated as separate from each other has been ongoing and has occurred at all levels, from the level of positive and negative affect as subdimensions of SWB to broader questions of whether well-being and its converse, ill-being, should themselves be treated as separable (Schimmack, 2008; Iasiello and Van Agteren, 2020; Zhao and Tay, 2023).

The results of our analysis suggest that the univariate and bivariate perspectives are both useful. Our earlier research of SWB (see Kaufman et al., 2022a) suggests that positive and negative aspects of well-being *can* be combined into an essentially unidimensional (or univariate) measure. Doing so is often desirable due to the versatility and comprehensibility of such an approach. However, the present study reveals key LPA shape differences by examining positive constructs (e.g., positive affect, life satisfaction) and negative affect separately (i.e., as bivariate dimensions). The debate over the structure of SWB will likely continue, but the present study suggests that the univariate and the bivariate approaches may each have their benefits, depending on context and research goals. While most researchers will likely be content with using a univariate approach, there may be cases where the additional insight provided by a bivariate approach is valuable—for example, if researchers’ aim is to maximize the explanation of variance in an outcome, or if they have a compelling theoretical reason to believe that the Ambivalent or Indifferent profiles relate in a unique way to a construct.

It is not difficult to imagine, for example, that those in the Indifferent profile may be over-represented in a population of patients exhibiting flat affect, while those in the Ambivalent profile may be over-represented in a sample of patients who have difficulties controlling their emotions (for example, those who are prone to both anger and excitement). While these are only examples, and need to be tested, the point is that there are compelling situations where researchers may prize the extra explanatory power offered by approaches that treat the positive and negative components of SWB as separable. And, finally, we note that this conceptualization of well-being aligns well with the theoretical ideal of happiness advanced by Diener and Biswas-Diener (2008), where the goal of individuals should not be to maximize positive emotion and eliminate negative emotion endlessly, but accept that the two will exist alongside each other, sometimes independent, sometimes balanced.

At the practical level, our present findings offer implications for practitioners who deal with well-being in clinical, educational, and organizational settings. First, our findings suggest that in real-world settings it may not be advisable to assume that individuals who present with high negative affect are necessarily lacking in the positive aspects of well-being. The presence of our Ambivalent profile suggests that a portion of the population experiences high levels of both positivity and negativity—and even more noteworthy, the Ambivalent profile also ranked second highest on our external criterion variable of Couple Satisfaction, suggesting that in at least one important area of life (relationships), some people can experience high levels of negativity and still rank highly on important aspects of well-being relative to their peers. Further research is needed to determine how far this principle can be generalized, but we note here that our findings echo previous researchers’ conclusions (e.g., Bateson et al., 2011) that reflexively seeking to reduce negativity without inquiring about its broader context is a sub-optimal clinical approach.

### 4.2 Limitations

Our research has limitations that we would encourage researchers to account for when interpreting our results and when designing future studies. First, our sample is representative of American coupled adults. Further research is needed to establish that our Indifferent and Ambivalent profiles have cross-cultural equivalents, estimate their prevalence, and determine how they relate to external criteria across cultures. We therefore caution against generalizing, but also suggest cross-cultural research as a potentially fruitful avenue for future exploration.

Second, LPA is methodologically distinct from more common, variable-centric forms of analysis. LPA is useful for identifying complex relationships between multiple variables by rendering them as groups (Spurk et al., 2020), but the Indifferent and Ambivalent profiles found here can also be explored using other approaches. In particular, SWB research may benefit if researchers explore bivariate scales that model positive and negative aspects of well-being as orthogonal dimensions, similar to the methods used by Rogge et al. (2017) to model relationship satisfaction. If successful, it would provide conceptual replication of the Ambivalent/Indifferent distinction found here, and may lead to the development of simpler ways to measure it.

A final limitation is our choice of dimensions. We accepted for the purposes of this analysis that the five subdimensions established in our previous research were sufficient as indicators. However, some researchers may prefer the original three dimensions recommended by Diener (1984), while others may wish to explore additional dimensions. Future research should use LPA to determine whether the profile solution found here replicates with different indicators. If so, it would further support the Ambivalent/Indifferent distinction.

### 4.3 Future directions

While the sample used in the present study is both large and nationally representative, the findings here still represent data from only a single study. A natural follow-up step to this research is to verify that this profile solution replicates when different measures of the dimensions of SWB are used (such as the PANAS or SPANE, for positive and negative affect), as well as replicating and providing further validation for the profile solution using other outcomes. In particular, we think it would be fruitful to examine our profile solution in relation to other forms of satisfaction such as job satisfaction, as well as consequential life outcomes such as depression, anxiety, perceived health, and flourishing. As a final note, it may be beneficial to expand the range of outcomes tested beyond measures of satisfaction and well-being to a wider range of constructs to determine if there are situations where the Ambivalent and Indifferent profiles are especially relevant. For example, in the present analysis the Ambivalent and Indifferent profiles predicted moderate-to-high levels of couple satisfaction, but it is possible that profiles like these, where levels of positivity and negativity are either muted or exaggerated, may produce markedly different results in how a person is perceived by their relationship partner. This may be a fruitful avenue for further investigation.

Our demographic table also hints at future research possibilities. The demographic information suggests that men are over-represented in the Indifferent profile while women are over-represented in the Ambivalent profile. While much research has been done on the relationship between SWB and demographic categories such as race, gender, and socioeconomic status, we think that there is also potential for research studying how demographic variables relate to our Ambivalent and Indifferent categories.

## 5 Conclusion

Researchers have long debated the structure of SWB. The present study offers a useful angle on this matter. LPA is prized for its ability to render complex relationships between multiple variables parsimoniously by representing them as groups (Spurk et al., 2020). These groups, in turn, likely reflect meaningful trends in the population. Here, the prevalence of two Ambivalent and Indifferent profiles, characterized by contrasting levels of positivity and negativity, suggest that there may be corresponding groups in the population whose experiences may not be easily captured by a univariate conceptualization of well-being where they are presumed to feel merely good or bad overall. While it is often useful to create univariate models of well-being, analyses like the present one are a reminder that researchers may sometimes benefit by adopting a bivariate conceptualization of SWB.

## Data availability statement

The raw data supporting the conclusions of this article will be made available by the authors, without undue reservation.

## Ethics statement

The studies involving humans were approved by University of California, Los Angeles – OHRPP. The studies were conducted in accordance with the local legislation and institutional requirements. The participants provided their written informed consent to participate in this study.

## Author contributions

CH: Conceptualization, Visualization, Writing – original draft, Writing – review & editing. LW: Conceptualization, Data curation, Investigation, Writing – review & editing, Writing – original draft. AR: Conceptualization, Formal analysis, Investigation, Methodology, Writing – original draft, Visualization. VK: Conceptualization, Data curation, Formal analysis, Investigation, Methodology, Project administration, Supervision, Writing – original draft, Writing – review & editing.
