# Supplementary material for: The diversity of well-being indicators: a latent profile analysis
Source: Front Psychol. 2024 Mar 4;15:1304074. doi: 10.3389/fpsyg.2024.1304074 (PMC10946337; doi:10.3389/fpsyg.2024.1304074)
Supplement: Supplementary file 1 [file Table_1.docx]

**Table S1**

*Model Fit Indices for Latent Profile Analyses*

| Model/  Solution | -2LL | AIC | BIC | aBIC | VLMRT | LMRT | Entropy |
| --- | --- | --- | --- | --- | --- | --- | --- |
| 1-Profile | 21099.62 | 21119.62 | 21172.66 | 21140.89 | — | — | — |
| 2-Profile | 18019.30 | 18051.30 | 18136.17 | 18085.35 | <.001 | <.001 | 0.92 |
| 3-Profile | 17335.01 | 17379.01 | 17495.71 | 17425.82 | 0.037 | 0.039 | 0.83 |
| 4-Profile | 16880.76 | 16936.76 | 17085.29 | 16996.34 | 0.017 | 0.018 | 0.84 |
| **5-Profile** | **16552.84** | **16620.84** | **16801.20** | **16693.19** | **0.039** | **0.041** | **0.85** |
| 6-Profile | 16280.68 | 16360.68 | 16572.86 | 16445.79 | 0.063 | 0.065 | 0.85 |

*Note.* -2LL=-2 Loglikelihood value; AIC=Akaike Information Criterion; BIC= Bayesian Information Criterion; aBIC=Adjusted Bayesian Information Criterion; VLMRT =Vuong-Lo-Mendell-Rubin Likelihood Ratio Test; LRMT=Lo-Mendell-Rubin Test. **Bold** values represent the best fitting model/solution.
