# Supplementary material for: The diversity of well-being indicators: a latent profile analysis
Source: Front Psychol. 2024 Mar 4;15:1304074. doi: 10.3389/fpsyg.2024.1304074 (PMC10946337; doi:10.3389/fpsyg.2024.1304074)
Supplement: Supplementary file 2 [file Table_2.docx]

**Table S2**

*Full Sample Bivariate Correlations*

|  | 1 | 2 | 3 | 4 | 5 | 6 |
| --- | --- | --- | --- | --- | --- | --- |
| 1. Domain Satisfaction | — |  |  |  |  |  |
| 2. Life Satisfaction | 0.75 | — |  |  |  |  |
| 3. Positive Affect | 0.59 | 0.59 | — |  |  |  |
| 4. Negative Affect | 0.49 | 0.50 | 0.47 | — |  |  |
| 5. Happiness | 0.61 | 0.62 | 0.69 | 0.66 | — |  |
| 6. Couple Satisfaction | 0.53 | 0.55 | 0.44 | 0.41 | 0.46 | — |

*Note.* Pearson zero-order bivariate correlations. All *p*s<.001. Negative affect was reverse scored so that higher scores indicate lower levels of negative affect.
